# Seen and Heard in Many Places

**Published:** 1898-11

**Authors:** 


### SEEN AND HEARD IN MANY PLACES.

THE announcement of the appointment of Dr. Rush Shippen Huidekoper as Chief Medical Director of the First Army Corps under Major-General Brooke, with the rank of lieutenant-colonel, has brought a keen thrill of satisfaction to many people in Philadelphia who know and admire him. He has made his home in

New York recently, under circumstances that are not entirely creditable to his native town of Philadelphia, and now that he has reached almost the pinnacle of his profession, there is a feeling of gratification among many who are glad to witness the spectacle of his distinguished preferment. His selection is due not merely to his knowledge of medicine, but to his standing as the leading veterinarian of America, and this statement contemplates no exception.

There are at least seven veterinary colleges and schools in this country—two in New York, one in Boston as an adjunct to Harvard University, one in Chicago, one in Minneapolis, one connected with Cornell University, and our own. Several of these can properly be demonstrated "diploma mills," their curriculum being limited and their term of matriculation brief. The majority, however, can present their graduates with parchments that entitle them to high rank in what has become a high profession. But of all these our own college, while it is the youngest, has already reached the highest phase of development and presents a degree of efficiency that, when its brief existence is considered, is simply amazing. For this result credit is very largely due to Dr. Huidekoper, although—and this is said in no spirit of harsh criticism—his labors do not appear to have been fairly and fully appreciated. Contentions arose which caused him to resign, and the New York college quickly captured him.

Dr. Huidekoper was graduated from the Medical Department of the University of Pennsylvania and became a regular practitioner. He displayed an aptitude for the treatment of young children. This he ascribed to his long-time liking for the cure of animals. Did it ever occur to you that the cure of both animals and babies must necessarily be very much alike? They both speak in a tongue unknown to the attendant, and neither can make their wants intelligible or describe the character of their complaint. The result is the doctor must diagnose the case in hand from what he can observe. Surely that requires a profounder knowledge and a deeper acumen than is requisite in the medical men who tell us grown folks that they know all about our internal condition. But I'm forgetting about Huidekoper. He found the ranks of the medical profession overcrowded and the chance for elevation to a scientific pinnacle very remote. In veterinary surgery, however, he found a vast field almost untilled. He saw there a rare opportunity for the enthusiastic scientist, and he seized it. He determined to become proficient in that kindred branch of the medical profession.

He first went to the famous school of Alfort, France, the cradle of veterinary surgery, and after his graduation studied among the learned men of Berlin When he returned home, discarding the offer of a professorship from Harvard University, he set vigorously to work to give his alma mater a veterinary department of which all good Philadelphians should be proud. Our University was willing to throw her protecting wing over the enterprise, but it would not expend one dollar to father it. In this dilemma the late J. B. Lippincott, the book publisher, and Joseph E. Gillingham, the lumber man, came to the rescue, the former contributing $30,000 and the latter $10,000. With this sum the handsome buildings in which the veterinary branch of the University has its location were erected. The grounds at Thirty-sixth and Pine Streets, which were donated by the city, occupy a space about two squares in extent. Even in their embryonic condition the buildings had a street frontage of over 250 feet, and comprised a commodious amphitheatre and museum, an anatomical or dissecting-room, a histological laboratory, a blacksmith shop with eight forges, a pharmaceutical laboratory, and several large stables for hospital purposes. The department's course of studies, which extends over three years, is rigid, comprehensive and exhaustive. It aims to give instruction, both theoretical and practical, in all branches pertaining to the scientific study of the elements of medicine, and the practical application of those elements to the domestic animals, in the preservation of their health, in their employment as useful aids to man, and in the diseases to which they are subject. In the second year the students are required to serve as aids in the hospital, and in the third year they are obliged to take charge of sick animals. This latter duty necessitates their taking turns at night watches, which require them to sleep in the buildings all night, except during the intervals when they are required to administer the proper medicine to some equine sufferer afflicted with the colic or some of the many other afflictions horses are subject to. To this institution, in its beginning, Dr. Huidekoper and his assistants gave tireless and unselfish attention.

The United States is far behind European countries in its recognition of the dignity and value of veterinary surgery. It is the only government in the world that does not in its army make a veterinary surgeon a commissioned officer. With us he cannot rise higher than a sergeancy, with a salary of $75 a month. One of the latest commissions appointed by the government for the purchase of horses for the army comprised a captain and a lieutenant of infantry and an officer of the commissary department. It did not even include a cavalry officer. Most folks think that the science of veterinary surgery concerns no matter of greater moment than the setting of a horse's broken leg. How ridiculous! As it is exemplified in our veterinary colleges it concerns every one of us intimately. One branch taught in the veterinary department of the University is termed zoötechnics, which is described in one of the official catalogues prepared by Dr. Huidekoper, when he was there, as including "the study of the laws of breeding and production of the domestic animal, heredity, race characteristics and individual impressions, the effect of climate, aliment, work, and the means to be employed in the selection and handling of animals to adapt them for the most economical profit, whether destined as motors, wool or milk-producers, or as articles of food." In other words, the science of veterinary surgery means instructing our sheep farmers in the best methods of wool production; it means the full utilization of horse labor; it means the prevention of pleuro-pneumonia, whereby millions of dollars are lost yearly to cattle-raisers, and the extermination of tuberculosis, whereby contaminated milk is given our children and actually kills thousands of them every year. Meaning all this, I am sure it means much and deserves substantial support.

Dr. Huidekoper once, in talking to the narrator, presented the importance of the work of the veterinary profession in a few striking words and figures. "In America," he said, "the advance in veterinary medicine has been far from keeping pace with our national reputation for energy and self-preservation. In 1806 Dr. Benjamin Rush, of the University of Pennsylvania, who had just been in Europe, and had seen the success of the institutions then a few decades old, wrote a letter to the Agricultural Society of Philadelphia and urged the importance of adding a veterinary department to the University. He called attention to the agricultural prospects of the country, and his letter was discussed before the society in 1807, but nothing practical was done. Our domestic animals have steadily increased in number and in value since that time. We had in 1852, horses, 5,000,000; cattle, 17,000,000; sheep, 22,000,000, swine, 30,000,000; value, $600,000,000; and in 1883 we had in the United States, horses, 10,838,111; value, $765,041,308; mules, 1,871,079.

In 1897 we had, horses, 14,364,667, valued at $452,649,396, a decrease from 1883, when we had 16,206,802, valued at $992,-225,185, a reduction in numbers and price, owing undoubtedly to the introduction of trolley and other electric motors. In the same year we had of mules 2,215,054, valued at $92,302,090, a reduction in number and value from the previous year, but not to a large extent; of milch cows we had 15,941,727, valued at $360,-239,993; of oxen and other cattle we had 30,508,408, valued at $507,929,421, a decrease of 7,000,000 from 1892 in number, but not an equal decrease in value; of sheep we had 36,818,643, valued at $67,020,942, a decrease of 11,000,000 from 1883 in number and a decrease in value of nearly $60,000,000; of swine we had 40,600,276, valued at $166,272,770, a decrease of 12,000,000 in number from 1892 and a decrease in value of about $75,000,000. These figures are given you to show the enormous value to this country of exact veterinary knowledge; and to Dr. Huidekoper more than to almost any other man is due the elevation of this science from its despised rut in the days of the blacksmith farrier and the country horse-doctor. The day of these has gone by. The veterinarian has taken rank with the oculist, gynecologist, the aurist, the dental surgeon, the neurologist, and the other specialists who remove the ills and soothe the ailments of afflicted humanity. When a man such as Huidekoper receives preferment in these war times it serves as an offset to some of President McKinley's dude military appointments.—MEGARGEE, in *Philadelphia Times*, May 31, 1898.

